# Parameter effect quantification for a phase change material-based lithium-ion battery thermal management system

**DOI:** 10.55730/1300-0527.3465

**Published:** 2022-07-08

**Authors:** Uğur MORALI

**Affiliations:** Department of Chemical Engineering, Faculty of Engineering and Architecture, Eskişehir Osmangazi University, Eskişehir, Turkey

**Keywords:** Battery thermal model, lithium-ion battery, phase change material, Taguchi design

## Abstract

The influence of discharge rate, ambient temperature, and phase change material on the maximum temperature and the highest temperature difference was investigated. The maximum temperature of the battery was tested with and without phase change material under extreme discharge rates (4C and 5C) and ambient temperatures (310 K and 320 K). Results showed that a phase change material reduced the maximum temperature from 327.94 K to 306.45 K for a 14.6 Ah lithium-ion battery discharged at 5C-rate and 320 K. Quantitatively determined parameter effects revealed that the PCM parameter considerably had a remarkable influence on maximum temperature compared to discharge rate and ambient temperature. Moreover, the influence of ambient temperature on the maximum temperature was approximately 2.5 times greater than the C-rate, while the influence of ambient temperature on the highest temperature difference was approximately 50 times greater than the C-rate. The quantified parameter effects can be used to improve the phase change material-battery cooling system.

## 1. Introduction

Lithium-ion batteries have been widely used in numerous applications due to their high energy density, high power density, and long cycle life [1, 2]. These features make the lithium-ion batteries promising candidates especially for portable devices, electric vehicles, and hybrid electric vehicles [3, 4]. However, charge/discharge operations generate heat inside the battery due to exothermic electrochemical reactions and conductive resistance [5, 6]. This heat generation increases the battery temperature. Manufacturers generally recommend that the safe operating temperature range for lithium-ion batteries should be between 20 and 40 °C [7]. In the literature, battery temperatures higher than 40 °C have been shown to influence battery performance adversely [8–10]. Thus, the lithium-ion battery temperature should be monitored and maintained at a safe operating temperature. Furthermore, keeping the temperature within the specific temperature range prevents the battery thermal runaway, fire, and explosion [11]. Moreover, battery temperature must be managed during battery operation, as temperature changes influence different processes in the thermal management system of electric vehicles. To achieve this goal, many researchers have investigated and studied various battery thermal management systems [12]. Different cooling techniques such as liquid cooling, air cooling, and phase change material cooling have been used to control the battery temperature [13]. Disadvantages associated with air/liquid cooling techniques are high power consumption and extra equipment such as liquid distribution systems and pump accessories. The phase-change material not only reduces the complexity of the cooling system but also ensures low production costs [12]. Furthermore, thermochemical properties such as high latent heat and wide operation temperature are advantages of phase change material-based cooling systems [14].

There are numerous parameters affecting temperature rise in lithium-ion batteries. Temperature rise, a major barrier for energy storage materials, is influenced by charge/discharge current and ambient temperature as well as cooling strategy. Lamrani et al. showed that when the ambient temperature increased from 25 °C to 35 °C, the maximum temperature of the lithium-ion battery increased by about 10.5 °C [15]. Therefore, battery temperature should be controlled more carefully in high ambient temperature environments. Another operating parameter, discharge rate, influences the temperature rise in lithium-ion batteries. High discharge rates may considerably increase the battery temperature due to generated heat based on the ohmic law [16]. Panchal et al. demonstrated that high ambient temperature and high discharge rates increased the temperature of both the cooling system and the battery [17]. This rise observed in battery temperature decreases the performance of batteries. Therefore, it is more significant to numerically and systematically investigate the effect of extreme conditions on temperature rise in lithium-ion batteries by implementing simulations. Many scientists have created thermal models to study the thermal properties of batteries [18, 19]. A faster comprehension of operating parameters that affect thermal behavior can be achieved [20]. The most widely used thermal models are Newman, Tiedemann, Gu, and Kim (NTGK) model, equivalent circuit model (ECM), and pseudo-two-dimensional (P2D) model. Compared to other models, ease of parameterization and computational simplicity made the NTGK model suitable to use in this work.

There have been numerous studies performed on PCM-battery thermal management in the open literature. Joshy et al. reported the vibration influence on PCM-based thermal management system of lithium-ion batteries at distinct discharge rates from 3C to 5C [21]. The frequency influence at low C-rates was more pronounced on temperature increase, while both amplitude and frequency at high C-rate showed a dominant effect on the temperature rise. Weng et al. proposed the thermal management of lithium-ion batteries by using a simple PCM structure [22]. They investigated the effect of different parameters such as PCM thickness, the temperature of phase change, and laying-aside time on the cooling performance. An et al. explored the thermal management of lithium-ion batteries at a discharge rate of 3C and ambient temperature of 40 °C by incorporating the composite PCM material combined with the liquid cooling system to show the effect of flow rate, channel arrangement, and PCM composition [14]. The cooling system with expanded graphite (6 wt%) decreased the maximum temperature by 2.1 °C. Jilte et al. designed a novel layout for a 18650 lithium-ion battery temperature control system using PCM to improve the cooling performance of each battery [23]. Better heat dissipation and uniform temperature distribution were obtained at different discharge rates (2C and 4C) and ambient temperatures (27, 35, and 40 °C). Verma et al. studied the battery pack temperature at different ambient temperatures (21 and 50 °C) by using capric acid as PCM [24]. The effect of distinct thickness of PCM (3, 7, 9, and 12 mm) on the battery temperature was investigated in which the 3 mm thickness of the PCM layer showed the best cooling performance. Zhang et al. prepared the composite PCM using kaolin, expanded graphite, and paraffin to manage the thermal features of a rectangular battery [25]. The single battery temperature could be maintained under 45 °C at 4C discharge rate with the optimum composition of PCM including 10 wt% expanded graphite, and 10 wt% kaolin. Furthermore, the temperature difference in a single battery did not exceed 5 °C owing to the thermal conductivity of the prepared PCM composite (6 W m^−1^ K^−1^). Celik et al. investigated the effect of ambient temperature (0 and 25 °C) and PCM features (amount and melting temperature) on a cylindrical lithium-ion battery used in a solar racing car [26]. The used PCM (melting temperature = 26 °C) exhibited a capacity rise of 3.15% owing to its thermal management properties. Although there have been extensive studies on battery thermal management in the literature, the effects of battery operating parameters have not been quantitatively determined. Because many studies of battery temperature do not include quantitative and statistical assessments of discharge parameter effects, the literature remains unclear about which discharge parameter was the most effective discharge factor for battery temperature. Therefore, there is a need for experimental design to quantify the parameter effect, which is difficult to obtain. If parameter effects can be determined quantitatively, it can be clearly stated which parameter should be controlled more carefully compared to other parameters. Therefore, parameter effect quantification was performed along with an electro-thermal model to design and improve robust battery thermal management systems. In this study, the thermal performance of a single battery and PCM cooled battery system was tested to evaluate the maximum temperature and highest temperature difference of lithium-ion batteries from engineering perspectives. The effect of ambient temperature (310 K and 320 K) and discharge rate (4C and 5C) on both maximum temperature and highest temperature difference (dT_max_) was determined numerically. The highest temperature difference was obtained by subtracting the minimum battery temperature from the maximum battery temperature. The relative significance of each parameter was compared quantitatively to present accurate parameter effects.

## 2. Materials and methods

Maximum temperature and dT_max_ of a prismatic lithium-ion battery were examined by using the NTGK model.

### 2.1. Materials

**Figure 1:**
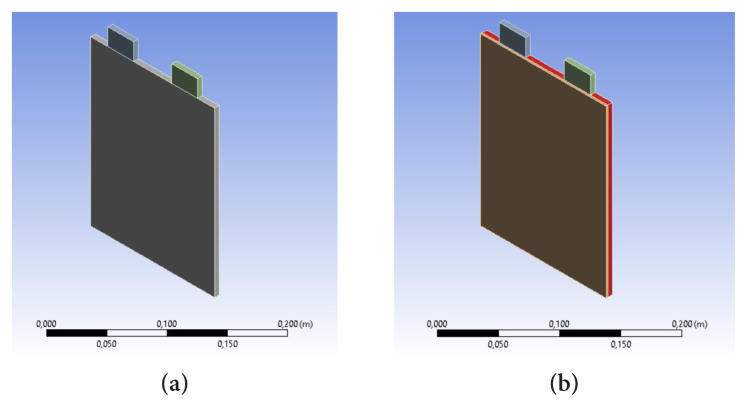
Configuration of lithium-ion battery system a) a single battery, b) phase change material-cooled battery.

A rectangular lithium-ion battery was used to numerically study the battery thermal behavior. The single lithium-ion battery configuration is displayed in Figure 1a. The main features of the battery are presented in Table 1. More details on the lithium-ion battery can be found in [27]. Figure 1b shows that 1 mm thickness phase change material was integrated with the XY plane. Paraffin RT44HC was used as a phase change material. The thermophysical features of PCM are summarized in Table 2.

### 2.2. Discharge procedure

**Figure 2:**
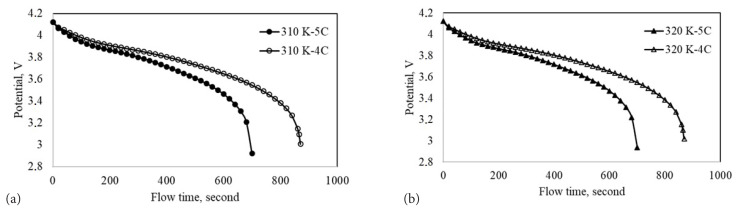
Discharge curves at 4C and 5C a) 310 K and b) 320 K.

The single battery with and without PCM was discharged at different C-rates and ambient temperatures to determine the effect of PCM on the maximum temperature and the highest temperature difference. Heat generation for a high capacity lithium-ion battery at 1C-rate discharge is not too high. Therefore, the temperature rise is not an issue for the low C-rates. The problem is generally at high C-rates. Much more heat is generated at high C-rates compared to low and moderate C-rates. Therefore, two discharge rates of 4C and 5C were chosen to evaluate the thermal behavior of the lithium-ion battery at two high ambient temperatures. The ambient temperatures selected were 310 and 320 K. The implemented discharge factors and their levels are shown in Table 3. The discharge curves are presented in Figure 2. Figures 2a and 2b shows the discharge curves at 310 and 320 K, respectively. The discharge protocols were created using two levels of three parameters. Orthogonal matrix indicating discharge procedures is presented in Table 4. Simulations were performed based on the L8(2^3^) orthogonal matrix. The maximum temperature and the highest temperature difference were obtained by simulating the discharge conditions presented in Table 4. For instance, the single battery was discharged at the ambient temperature of 310 K and discharge rate of 4C according to L1 discharge protocol. Similarly, the PCM cooled battery was discharged at 310 K and 4C-rate by following L2 discharge protocol. The maximum temperature and the highest temperature difference were evaluated as response variables according to the Taguchi design. It is important to note here that the aim of this design is the minimization of the maximum temperature and the highest temperature difference. Therefore, the smaller-is-better assessment was selected as the quality characteristic to calculate the signal-to-noise ratio (S/N). The S/N ratios calculated were used to build the response table for the response variables and calculate the corresponding delta values. Moreover, the delta values were used to rank the discharge parameter effects.

### 2.3. Governing equations

The energy equation of the single lithium-ion battery can be modeled by Equation 1:


(1) $$\frac{\partial }{\partial t}\left(\rho C_{p}T\right)=\nabla (k\nabla T)+Q_{gen}$$

where ρ and *C_p_* are density and specific heat capacity, respectively. *T* denotes temperature, *t* shows time, *Q_ge_*_n_ indicates the heat generated by the battery.

The equation of generated heat can be expressed as:


(2) $$Q_{gen}=I\left(U_{ocp}-U\right)+I(T\frac{dU_{ocp}}{dT})$$

where *I* is applied current, *U_ocp_* is open circuit potential, *U* is battery potential, (*dU_ocp_/dT*) is entropic heat coefficient. *Q_gen_* presented in Equation 2 considers both reversible and irreversible heat. The entropic heat can be neglected since the Joule heating is the main source of heat generation compared to reversible heat.

The Newman, Tiedemann, Gu, and Kim (NTGK) model was applied in this study because it is a simple quasiexperimental electro-thermal model. The NTGK model was parameterized using a discharge curve obtained at a certain C-rate. The NTGK model is given by Equations 3–5:


(3) $$\nabla (\alpha_{+}\nabla \gamma_{+}=-(j_{ECh}-j_{short})$$

(4) $$\nabla (\alpha_{-}\nabla \gamma_{-}=j_{ECh}-j_{short}$$

(5) $$j_{ECh}=\alpha \text{Y }[U_{ocp}-(\gamma_{+}-\gamma_{-})]$$

where α*_+_* and α*_−_* are the electric conductivity of cathode and anode, γ_+_ and γ*_−_* are cathodic phase potential and anodic phase potential. *j_ECh_* shows current transfer rate per unit volume and *j_short_* denotes current transfer rate. *U* and *Y* parameters as function of depth-of-discharge (DOD) are presented as follows [28–30]:


(6) $$U=\alpha_{0}+a_{1}(DOD)+a_{2}{\left(DOD\right)}^{2}+a_{3}{\left(DOD\right)}^{3}$$

(7) $$Y=a_{4}+a_{5}(DOD)+a_{6}{\left(DOD\right)}^{2}$$

where is the fitting parameters. The fitting parameters calculated in [31] were used in this study.

### 2.4. Phase change material model

The heat transfer in PCM was simulated by using the enthalpy change method. The energy equation of PCM is given by Equation 8:


(8) $$\rho_{PCM}\frac{\partial H}{\partial t}=k_{PCM}\nabla^{2}T$$

where ρ*_PCM_* is density, *k_pcm_* is thermal conductivity. The specific enthalpy H can be expressed as:


(9) $$H=h_{s}+\Delta H$$

(10) $$h_{s}=h_{0}+\int_{T_{0}}^{T}C_{p,PCM}dT$$

(11) ΔH=θL

where *h_s_* shows sensible enthalpy, ∇H presents melted PCM enthalpy, *h_0_* denotes the reference enthalpy at temperature *T_0_*. Liquid fraction, θ=0, if *T* < *T_solidus_* and θ=1, if *T* > *T_liquidus_*.

### 2.5. Computational procedure and meshing

**Figure 3:**
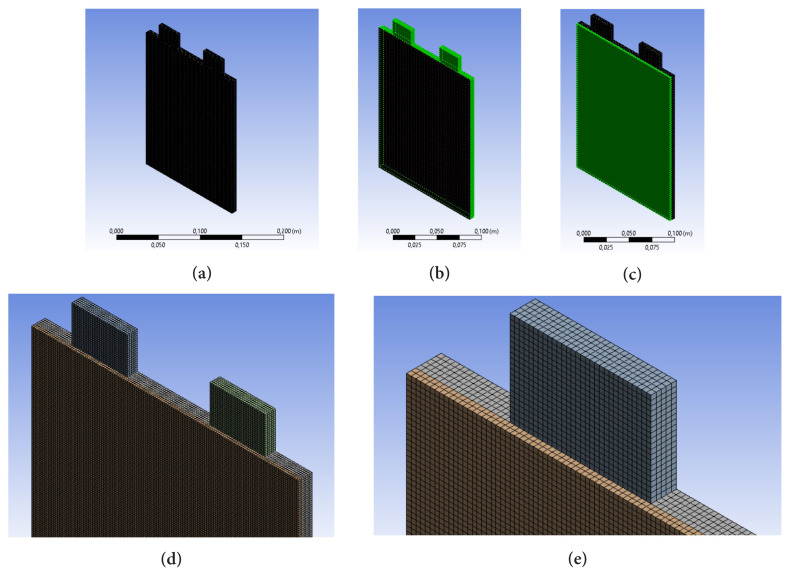
Mesh structure of lithium-ion battery and phase change material a) meshing of the computational domain, b) meshing of PCM, c) meshing of lithium-ion battery, d and e) the high resolution meshing of the domain.

The thermal model of the lithium-ion battery was solved through the NTGK model. ANSYS Workbench was used to set a 3D model. Figure 3a shows the meshing of the computational domain. Schematic of the meshing of one-side PCM cooled battery is shown in Figures 3b (meshing of PCM) and 3c (meshing of lithium-ion battery). The high-resolution meshing is also presented in Figures 3d and 3e. The initial temperature of the PCM-cooled battery system was assumed to be equal to 300 K. The battery convective heat transfer coefficient was assumed to be equal to 5 W m^−1^ K^−1^. Convective heat transfer between the PCM and the ambient with a coefficient of 2.25 W m^−1^ K^−1^ was also considered. The heat was transferred between the battery and PCM inner surfaces by the conduction mechanism. The radiation heat transfer was neglected.

**Figure 4:**
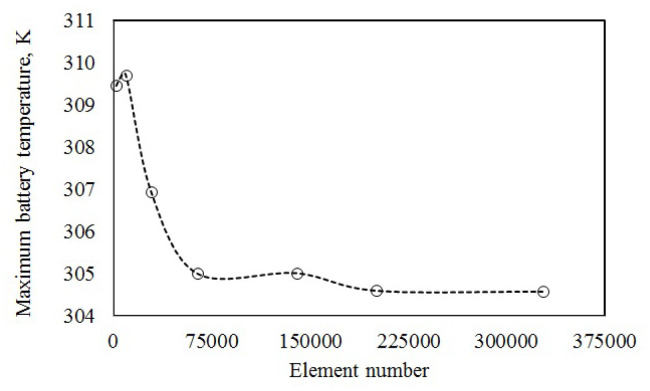
Grid independence analysis.

A grid independence test was performed to show that results were element number-independent. A tolerance level of 0.1% was used to evaluate the association between the maximum temperature and element number. Figure 4 presents the relationship between the element number and maximum temperature. Maximum temperature was obtained as 304.6079 K for element number 200880 and as 304.5864 K for element number 327304. When the element number reaches 200880, the maximum temperature changes slightly with increasing element number, and this appears to be stable for further numerical processing. The change in element number showed a change less than 0.1% of the maximum temperature with increasing element number from 200880 to 327304. The element number of 200880 both reduced the time spent for simulation and did not affect the accuracy of the results. Therefore, it was selected for the subsequent simulations.

**Figure 5:**
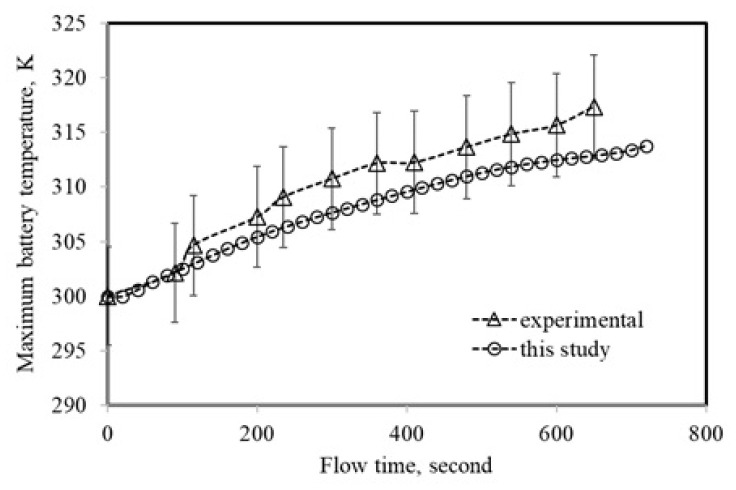
Validation of the NTGK model for maximum temperature at 5C-rate.

The maximum temperature of the battery discharged at 5C-rate was numerically obtained and was validated with the experimental test results carried out by [31]. The experimental results were matched with the simulated maximum temperatures. The validity of the confirmation results is shown in Figure 5. The NTGK model predicted the maximum temperature with good accuracy, as Figure 5 demonstrates that the experimental and theoretical temperatures align within 2% at all flow times. Thus, it was demonstrated that the battery model was reliable for subsequent numerical studies.

## 3. Results and discussion

Quantification of the parameter effects on the maximum temperature and the highest temperature difference was done by the Taguchi design.

### 3.1. Maximum temperature

**Figure 6:**
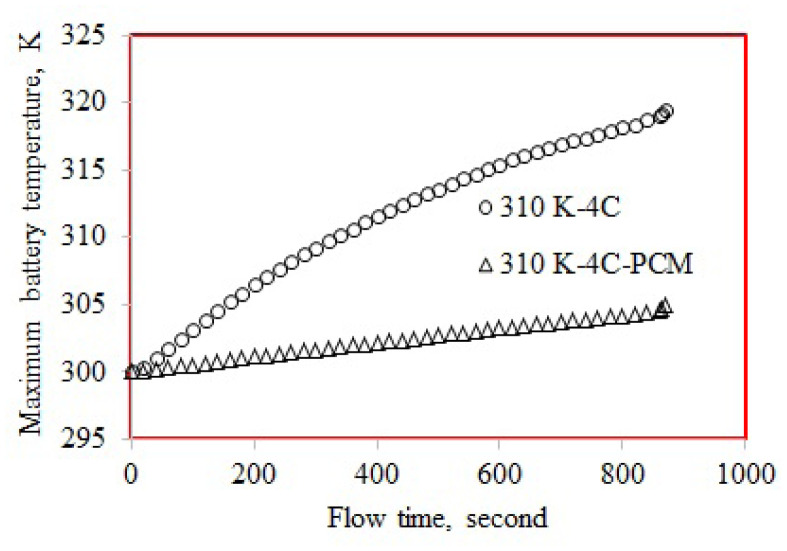
Maximum temperature at 4C-rate and 310 K.

**Figure 7:**
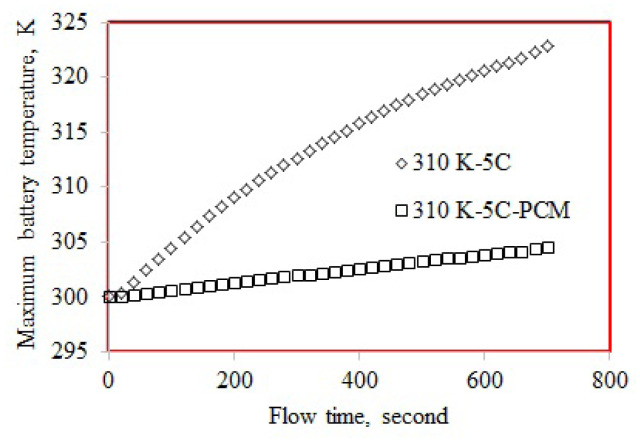
Maximum temperature at 5C-rate and 310 K.

Table 5 shows the numerically obtained maximum temperatures. The battery exhibited the highest maximum temperature of 327.94 K at 320 K and 5C-rate. The lowest maximum temperature was 304.61 K and calculated for the battery discharged at 310 K and 5C-rate. The maximum temperature obtained at 4C and 310 K is presented in Figure 6. The maximum temperature was 319.42 K for the battery discharged at 310 K and 4C-rate, while it was 304.90 K for the PCM cooled battery. The maximum temperature was 14.52 K lower for the PCM cooled battery. Figure 7 displayed the maximum temperature for the battery discharged at 5C-rate (ambient temperature of 310 K). Concerning 310 K ambient temperature, the maximum temperature obtained at 5C (322.84 K) was higher than that at 4C (319.42 K). The maximum temperature of PCM cooled battery discharged at 5C-rate was 304.61 K obtained at 310 K ambient temperature. Regarding the 310 K ambient temperature, the maximum temperature of the PCM cooled battery discharged at 5C was 18.23 K lower than the battery discharged without PCM. The cooling performance of PCM at 5C was 18.23 K, while it was 14.52 K at 4C-rate. To interpret this result, the maximum temperatures obtained for the single battery cells can be evaluated. The maximum temperature of the single battery discharged at 5C-rate was higher than that at 4C-rate. Therefore, the temperature difference between the PCM and battery cell discharged at 5C-rate was higher than that at 4C-rate.

**Figure 8:**
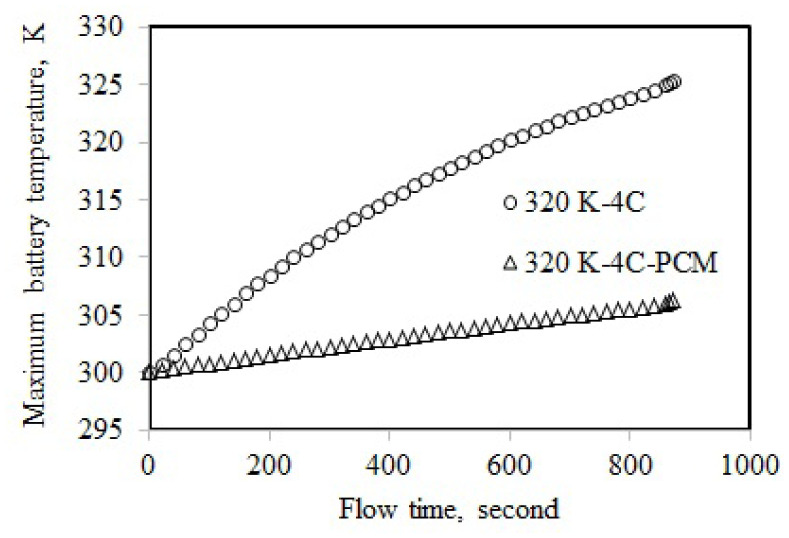
Maximum temperature at 4C-rate and 320 K.

**Figure 9:**
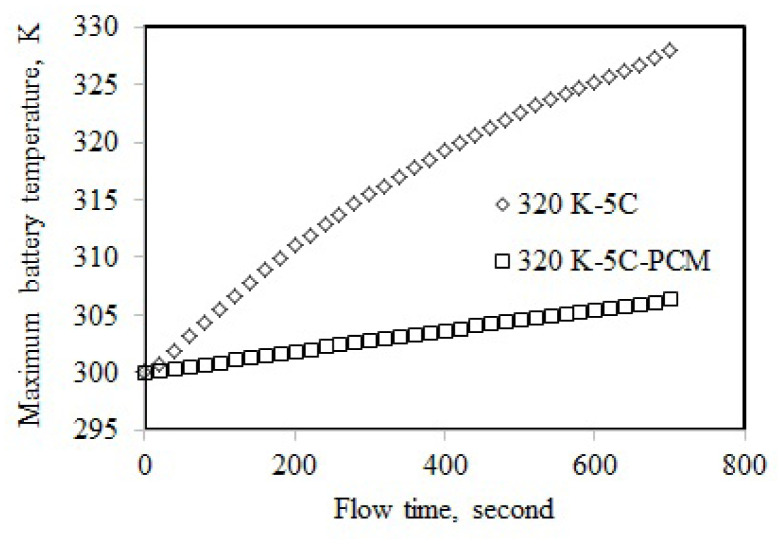
Maximum temperature at 5C-rate and 320 K.

The maximum temperature of the single battery cell was 325.36 K at 4C-rate discharge when the ambient temperature was 320 K (Figure 8). The maximum temperature was decreased using the PCM cooling by 18.99 K, and it was 306.38 K. The maximum temperature for L5 was higher than that of L1. This could be attributed to the effect of the higher ambient temperature of L5 compared to L1. The maximum temperatures obtained at 5C-rate and 320 K are displayed in Figure 9. The maximum temperature of L7 was 327.94 K, while it was 306.45 K for L8, implying a cooling of 21.48 K. The highest cooling performance was obtained at 5C and 320 K. This could be attributed to the highest maximum temperature of 327.94 K for the single battery cell. The maximum temperature of the single battery discharged at 5C was decreased from 327.94 K to 322.84 K with a decrease in the ambient temperature from 320 K to 310 K. This decrease showed the influence of ambient temperature on the maximum temperature. The battery discharged at 5C was cooled to 304.61K using PCM when the ambient temperature was 310 K.

### 3.2. Quantification of parameter effect

It is difficult to perform a huge number of experiments at abuse conditions that cause time consumption; complete statistical analysis could enable both faster evaluation of temperature behavior and quantitative analysis of battery temperature. The Taguchi design was used to extract the maximum amount of information from the minimum number of runs. The S/N ratio was used to identify the control parameter settings that minimize the variability caused by the noise parameters. The S/N for each combination of control factor levels in the design was calculated. The smaller-is-better was selected depending on the goal of our experiment. The S/N ratios calculated based on the smaller-is-better quality characteristic using the maximum temperatures in Table 5 are presented in Table 6. The lowest S/N ratio was calculated for L7, while the highest S/N ratio was obtained for L4. The response table was tabulated using the S/N ratios in Table 6 to calculate the S/N ratio for each level of discharge parameters. The response table consists of delta, rank, and S/N ratios. Delta was the difference between the S/N ratio of the levels of each parameter. Delta can be interpreted as a measure of relative parameter effect. Table 7 presents how the effect of each parameter varies with parameter levels. The highest delta value was 0.5049 and was obtained for the PCM parameter. The lowest delta value was 0.0386 and was obtained for the C-rate. The delta value was 0.0976 for the ambient temperature, and it was lower than the PCM. It is important to emphasize here that the delta value was used to quantify the effect of each discharge parameter. Note also that changes in the delta value ultimately reflect changes in the discharge parameter effects. Delta values also showed that the influence of PCM on maximum temperature was approximately 5 times greater than the ambient temperature. The results showed that the influence of the C-rate parameter on the maximum temperature was approximately 40% that of the ambient temperature. Rank values in Table 7 also indicated the relative effect of the parameters on the maximum temperature. According to the rank values, the highest effect was obtained for the PCM, while the C-rate exhibited the lowest influence on the maximum temperature. The results showed that decreased C-rate could improve temperature rise at the end of discharge, but not as much as might be expected. The PCM parameter was the most significant factor to minimize the maximum temperature.

The S/N ratios were calculated for the highest temperature difference to determine the influence of discharge parameters on the highest temperature difference (Table 8). The highest temperature difference was at its maximum value for the battery discharged at 5C and 310 K (without PCM), as expected. The highest temperature difference for that of PCM cooled battery was 0.18, the second-lowest highest temperature difference. The lowest highest temperature difference was 0.16 and was obtained for the PCM cooled battery discharged at 320 K and 5C-rate. The highest temperature difference was 2.27, and it was calculated for the battery discharged at 5C and 320K (without PCM). At 4C-rate, the highest temperature difference of the battery discharged at 310 K (1.66) was higher than that at 320 K (1.44). The highest temperature difference for L6 was 0.24. The highest temperature difference was 0.28 for the battery discharged at 310 K and 4C-rate using PCM cooling. The highest temperature difference of L2 was higher than the L4. This could be attributed to the influence of the C-rate. The battery discharged at a greater C-rate had a lower time to transfer the heat from the battery.

**Figure 10:**
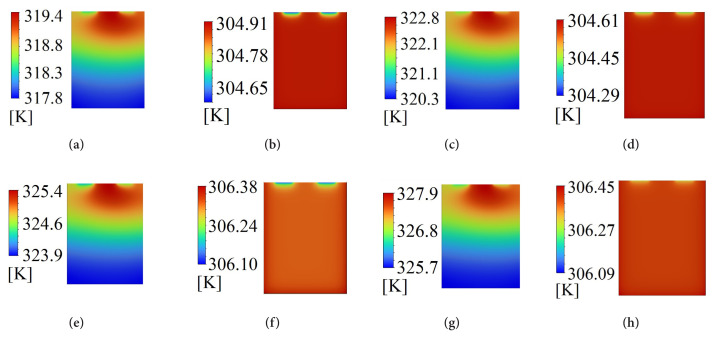
Temperature counters of lithium-ion battery obtained for discharge conditions: a) L1, b) L2, c) L3, d) L4, e) L5, f) L6, g) L7, h) L8.

Figure 10 shows the temperature counters of each battery discharged based on the L8 orthogonal array. The temperature counters in Figures 10a–10h displayed the generated heat accumulated in the upper center of the cell (without PCM), indicated by higher temperatures. Alternatively, more heat was transferred from the battery bottom (without PCM). Irrespective of the ambient temperature and C-rate, uniform temperature distribution was developed for each PCM cooled battery. Moreover, the lowest temperature for the PCM cooled batteries was observed at the tab zones connections. The heterogeneous temperature distribution was more evident in the L3 condition as compared to the other conditions. The heterogeneous temperature distribution for the L3 condition combinedly manifested as high maximum temperatures and low minimum temperatures. When combined with the maximum battery temperature and the corresponding minimum battery temperature (Figures 10g and 10h), it can be concluded that the battery was able to maintain its uniform temperature distribution even at 5C-rate by using PCM.

The S/N ratio of each level of each discharge parameter for the highest temperature difference was calculated using the S/N ratios in Table 8. The calculated S/N ratios were used to obtain delta values of each discharge parameter. The delta values are presented in Table 9. The highest delta value of 19.278 was obtained for the PCM parameter, revealing that the PCM parameter was the most significant factor to the highest temperature difference. The delta value of ambient temperature was smaller than the PCM parameter and higher than the C-rate. The influence of ambient temperature on the highest temperature difference was lower than the PCM parameter. The results showed that a slight change in ambient temperature could significantly contribute to a larger temperature difference compared to the C-rate. The results also demonstrated that the influence of ambient temperature was approximately 50 times the effect of the C-rate. The lowest delta value of 0.023 was obtained for the C-rate parameter, meaning that other discharge parameters were more important in affecting the highest temperature difference. The results showed that the PCM and ambient temperature were found to be the two dominating discharge parameters for the batteries examined in this work. However, the C-rate should not be completely overlooked as the main factor.

## 4. Conclusion

This study presented the maximum temperature behavior of lithium-ion batteries at multiscale multidimension, combining electrothermal simulation and statistical analysis. The impact of discharge parameters for the lithium-ion battery with and without PCM cooling was quantitatively determined. The highest maximum temperature of 327.94 K was obtained for the battery discharged at 320 K and 5C-rate and decreased down to 306.45 K by employing the phase change material. The highest maximum temperature was 322.84 K (ambient temperature = 310 K and 5C-rate) and was decreased to 304.61 K with PCM cooling. PCM cooling exhibited a more pronounced effect on each response variable compared to ambient temperature and C-rate. The influence of ambient temperature on the maximum temperature was approximately 2.5 times greater than the C-rate, while the influence of ambient temperature on the highest temperature difference was approximately 50 times higher than the C-rate. For the phase change material-cooled battery, more consideration should be given to the ambient temperature compared to the C-rate. Quantitatively presented parameter influences can improve the phase change material-based battery thermal management systems by indicating the significance of each parameter. This study calls for more statistical methods to quantify battery temperature and correlate it with different charge/discharge parameters.

## Figures and Tables

**Table 1:** Main battery parameters.

Specification	Value
Positive electrode	Manganese oxide-based
Negative electrode	Graphite
Electrolyte	Polymer based
Nominal capacity, Ah	14.6
Maximum voltage, V	4.2
Minimum voltage, V	3.0
Dimensions, cm	19.20 × 14.50 × 0.54

**Table 2:** Thermal and physical properties of phase change material.

Parameter	Value
Density (solid)	880 kg m^−3^
Density (liquid)	770 kg m^−3^
Specific heat capacity	2250 J kg^−1^ K^−1^
Thermal conductivity (solid)	0.21 W m^−1^ K^−1^
Thermal conductivity (liquid)	0.18 W m^−1^ K^−1^
Dynamic viscosity	0.02 kg m^−1^ s^−1^
Latent heat	270700 J kg^−1^
Solidus temperature	314.15 K
Liquidus temperature	317.15 K

**Table 3:** Level of discharge parameters.

Level	Parameter		
	Ambient temperature, K	C-rate, h^−1^	Cooling
1	310	4	PCM−
2	320	5	PCM+

**Table 4:** Discharge protocol based on the L8 orthogonal design.

Run	Ambient temperature, K	C-rate, h^−1^	Cooling
L1	310	4	PCM−
L2	310	4	PCM+
L3	310	5	PCM−
L4	310	5	PCM+
L5	320	4	PCM−
L6	320	4	PCM+
L7	320	5	PCM−
L8	320	5	PCM+

**Table 5:** Maximum temperature for each run presented in Table 4.

Run	Maximum temperature, K
L1	319.42
L2	304.90
L3	322.84
L4	304.61
L5	325.36
L6	306.38
L7	327.94
L8	306.45

**Table 6:** Signal to noise ratios of maximum temperature.

Run	Signal to noise ratio, dB
L1	−50.09
L2	−49.68
L3	−50.18
L4	−49.67
L5	−50.25
L6	−49.73
L7	−50.32
L8	−49.73

**Table 7:** Response table for maximum temperature.

Level	Ambient temperature	C-rate	PCM
Level 1	−49.906	−49.936	−50.208
Level 2	−50.004	−49.974	−49.703
Delta	0.0976	0.0386	0.5049
Rank	2	3	1

**Table 8:** Signal to noise ratios of the highest temperature difference.

Run	Highest temperature difference, K	Signal to noise ratio, dB
L1	1.66	−4.40
L2	0.28	11.11
L3	2.50	−7.96
L4	0.18	14.90
L5	1.44	−3.19
L6	0.24	12.43
L7	2.27	−7.11
L8	0.16	16.02

**Table 9:** Response table for the highest temperature difference.

Level	Ambient temperature	C-rate	PCM
Level 1	3.413	3.985	−5.665
Level 2	4.535	3.962	13.613
Delta	1.123	0.023	19.278
Rank	2	3	1
